# Correction: Amyloid fibrillation of the glaucoma associated myocilin protein is inhibited by epicatechin gallate (ECG)

**DOI:** 10.1039/d2ra90131e

**Published:** 2022-12-23

**Authors:** Ritika Sharma, Anchala Kumari, Bishwajit Kundu, Abhinav Grover

**Affiliations:** School of Biotechnology, Jawaharlal Nehru University New Delhi-110067 India sharma.ritika198@gmail.com abhinavgr@gmail.com +91-8130738032; Indian Council of Medical Research, International Health Division New Delhi-110029 India anchala.choudhary27@gmail.com; Kusuma School of Biological Sciences, Indian Institute of Technology Delhi Hauz Khas New Delhi India – 110016 bkundu@bioschool.iitd.ac.in

## Abstract

Correction for ‘Amyloid fibrillation of the glaucoma associated myocilin protein is inhibited by epicatechin gallate (ECG)’ by Ritika Sharma *et al.*, *RSC Adv.*, 2022, **12**, 29469–29481, https://doi.org/10.1039/D2RA05061G.

In the original article, in Section 4.11 REMD simulations, the number of replicas, their average exchange probability and the temperature range utilized were inaccurately reported as 32, 20% and 290–500 K, respectively. However, the presented results were obtained using 48 replicas, with an average exchange probability of 13% and a temperature range of 298–380 K. The authors regret this error. The scientific conclusions of this article remain unchanged.

The Royal Society of Chemistry apologises for these errors and any consequent inconvenience to authors and readers.

## Supplementary Material

